# Enhanced beta-1 adrenergic receptor responsiveness in coronary arterioles following intravenous stromal vascular fraction therapy in aged rats

**DOI:** 10.18632/aging.102069

**Published:** 2019-07-11

**Authors:** Gabrielle Rowe, Natia Q. Kelm, Jason E. Beare, Evan Tracy, Fangping Yuan, Amanda J. LeBlanc

**Affiliations:** 1 Cardiovascular Innovation Institute, University of Louisville, Louisville, KY 40292, USA; 2 Department of Physiology, University of Louisville, Louisville, KY 40292, USA; 3 Kentucky Spinal Cord Injury Research Center, University of Louisville, Louisville, KY 40292, USA

**Keywords:** aging, cell therapy, coronary, adrenergic, vasodilation

## Abstract

Our past study showed that a single tail vein injection of adipose-derived stromal vascular fraction (SVF) into old rats was associated with improved dobutamine-mediated coronary flow reserve. We hypothesize that i.v. injection of SVF improves coronary microvascular function in aged rats via alterations in beta adrenergic microvascular signaling. Female Fischer-344 rats aged young (3 months, n=32) and old (24 months, n=30) were utilized, along with two cell therapies intravenously injected in old rats four weeks prior to sacrifice: 1x10^7^ green fluorescent protein (GFP+) SVF cells (O+SVF, n=21), and 5x10^6^ GFP+ bone-marrow mesenchymal stromal cells (O+BM, n=6), both harvested from young donors. Cardiac ultrasound and pressure-volume measurements were obtained, and coronary arterioles were isolated from each group for microvessel reactivity studies and immunofluorescence staining. Coronary flow reserve decreased with advancing age, but this effect was rescued by the SVF treatment in the O+SVF group. Echocardiography showed an age-related diastolic dysfunction that was improved with SVF to a greater extent than with BM treatment. Coronary arterioles isolated from SVF-treated rats showed amelioration of the age-related decrease in vasodilation to a non-selective β-AR agonist. I.v. injected SVF cells improved β-adrenergic receptor-dependent coronary flow and microvascular function in a model of advanced age.

## Introduction

Coronary perfusion, reflective of coronary vascular function, is compromised by as much as 43% in advanced age [1] and can contribute to the prevalence of cardiovascular diseases (CVDs) such as heart failure (HF) and coronary microvascular disease (CMD). There are several neurohormonal mechanisms that are activated in order to maintain resting cardiac output (CO) in aging and HF, including sympathetic overdrive [2]. As a result of this overactivation, a desensitization/downregulation of cardiac β-adrenergic receptors (β-AR) occurs over time which leads to decreased cardiac contractility and inotropic reserve [3,4]. This age-related decrease in catecholamine-responsiveness in the elderly, as well as a decrease in β-AR vasorelaxation in both animals and humans [5–7], results in a shift away from coronary vasodilation and toward vasoconstriction [5].

In a healthy human heart, there is a 4:1 ratio of β1:β2-AR, with minimal expression of β3-AR [8,9]. All three β-ARs are associated with the stimulatory G protein (Gαs) activation and can have both stimulatory and inhibitory effects on the heart by altering inotropy, lusitropy, and chronotropy [10,11]. While β1-AR is present in all cardiomyocytes, β2-AR is more abundant in endothelial cells and vascular smooth muscle cells (VSMC) throughout the body [12,13], while β3-AR is primarily expressed in white and brown adipose tissue [14,15]. Small coronary resistance arterioles, which determine appropriate increases in vascular blood flow (BF) and can lead to CMD when dysfunctional, exhibit a predominance of β2-AR [16]. Regardless, an age-associated decrease in β-AR sensitivity and density, which is consistent across the species, has been shown in cardiac muscle [17] and has mostly been attributed to a downregulation of β1-AR [18]. However, it’s unknown if β1- and β2-AR signaling in coronary microvessels is altered with age. Since β-AR signaling is abnormal in failing hearts as well as in aged hearts, this pathway is a desirable diagnostic and therapeutic target.

Cell therapies such as mesenchymal stem/stromal cells (MSCs) are thought to act by delivering paracrine factors that promote angiogenesis and modulate inflammation within the treated tissue [19–22]. Our laboratory has recently described how an intravenous (i.v.) injection of adipose-derived stromal vascular fraction (SVF) was associated with improved dobutamine-mediated coronary flow reserve (CFR) and diastolic function in aged rats *in vivo* compared to old control rats and those injected with an endothelial cell population [23]. Even though we identified incorporated green fluorescent protein (GFP+) SVF cells in the cardiac and coronary vascular tissue upon explant, isolated coronary vasoreactivity to endothelin, bradykinin, and increases in pressure did not reveal group differences in this study, which led us to explore changes in adrenergic-specific signaling following SVF injection. Previously, MSCs have been shown to influence adrenergic signaling in two models. In 2006, intramyocardial injection of bone-marrow MSC (BM-MSC) led to β-AR upregulation and improved cardiac contractility in a model of non-ischemic HF in rabbits [24], while a more recent study described the rescue of cardiac function under adrenergic challenge in diabetic rats after i.v. injection of BM-MSCs with concomitant restoration of β1-AR mRNA expression in the left ventricle (LV) [25]. However, it is not known if either BM-MSC or SVF cell therapies restore adrenergic signaling in the heart and coronary microvasculature in a model of advanced age, and if this occurs through similar mechanisms.

Therefore, based on the gaps in conclusions from the aforementioned studies, our purpose was to determine if SVF and/or BM-MSC improve beta-adrenergic signaling in the aged heart. Our hypothesis was that cell therapy (either BM-MSC or SVF) will improve β1- and β2-AR-mediated vasodilation in coronary arterioles and this will be associated with improved CFR in aged rats. We will test this hypothesis by comparing young, old, and old rats injected intravenously with either BM-MSC or SVF and evaluate cardiac and microvascular function using ultrasound, pressure-volume (PV) loops, pressure myography, and immunofluorescence focusing on adrenergic-specific signaling.

## RESULTS

### Animal characteristics and circulating catecholamines

To determine if systemic i.v delivery of SVF or BM-MSC affected fundamental animal characteristics and circulating plasma catecholamine levels, we collected gross anatomical measurements and sampled right ventricular blood; these data are presented in Table 1. All aged groups (OC, O+SVF, and O+BM) exhibited increased body weight (BW), total ventricular weight, and LV weight compared to YC (Table 1). Although it was not significant, there were higher circulating plasma levels of norepinephrine (NE) and epinephrine (EPI) in OC rats than in YC and O+SVF (Table 1). The O+BM group had significantly lower NE (0.2±0.03, n=6) and EPI (0.05±0.01, n=6) levels compared to OC (NE 3.32±0.96; EPI 2.12±0.5, n=15) (Table 2). There were no differences in plasma levels of serotonin (5HT) between the groups. The OC (0.12±0.02, n=15) showed significantly higher dopamine (DA) levels compared to YC (0.04±0.01, n=10) (Table 1).

**Table 1:** Animal characteristics and plasma catecholamine levels.

**Animal Characteristics**	**YC (n=26)**	**OC (n=26)**	**O+SVF (n=20)**	**O+BM (n=6)**
Age (months)	4.24±0.09	24.99±0.17	24.66±0.22	25.42±0.26
Body weight (g) (n)	179.95±3.26 (21)	250.04±4.95 * (24)	246.35±9.84 *	264.8±7.39 *(5)
Total Ventricular weight (mg) (n)	49.61±1.39 (18)	65.62±1.38 * (22)	63.59±0.97 * (18)	68.4±3.34 *
LV weight (mg) (n)	38.97±1.39 (18)	54.55±1.81 * (22)	49.73±2.4 * (18)	53.97±1.18 *
**Plasma Catecholamine Levels (ng/mL)**	**YC (n=10)**	**OC (n=15)**	**O+SVF (n=5)**	**O+BM (n=6)**
Norepinephrine (NE)	0.5±0.14	3.32±0.96	0.27±0.04	0.20±0.03 #
Epinephrine (Epi) (n)	0.52±0.16	2.12±0.5	0.05±0.01	0.05±0.01 #
Serotonin (5HT) (n)	35.99±12.23	139.39±43.38	69.07±35.9	44.02±15.34
Dopamine (DA) (n)	0.04±0.01	0.13±0.02 *	0.05±0.01	0.07±0.01

Gross anatomical measurements and catecholamine levels were measured and averaged. *P* ≤ 0.05 vs Young Control (*), vs Old Control (#), and vs Old+SVF ($); data are presented as means±SEM (n) and analyzed with one-way ANOVA followed by *post hoc* tests where appropriate.

**Table 2:** Summary of cardiac functional parameters during echocardiography and PV loop recordings.

**Ultrasound Parameter**	**YC (n=10)**	**OC (n=9)**	**O+SVF (n=10)**	**O+BM (n=6)**
Heart Rate (BPM)	334±19	342±39	322±21	325±21
LVDs (mm)	1.92±0.2	2.17±0.33	2.58±0.44 * #	3.23±0.47 * # $
LVDd (mm)	4.85±0.34	5.2±0.31	5.49±0.38 *	5.50±0.25 *
LVVs (µL)	11.89±3.26	16.51±5.99	25.74±9.88 *	43.61±14.64 * #
LVVd (µL)	111.48±17.35	130.83±17.9	148.29±23.11 *	148.23±15.73 *
Stroke Volume (µL)	99.58±16.49	114.33±17.26	122.56±17.10 *	104.63±15.49 # $
Ejection Fraction (%)	89.31±2.93	86.16±4.41	85.042±3.86	75.25±6.08 * # $
Fractional Shortening (%)	60.51±3.96	56.99±6.08	56.21±4.65	44.68±5.21 * # $
Cardiac Output (mL/min)	36.31±4.57	38.75±4.54	39.35±5.02	37.37±1.86
LV Mass (mg)	418.92±46.44	460.53±89.22	500.49±46.39 *	498.04±27.31
**Hemodynamic Parameter**	**YC (n=13)**	**OC (n=16)**	**O+SVF (n=12)**	**O+BM (n=6)**
Heart Rate (BPM)	223±11	175±7	184±9	192±10
Systolic BP (mmHg)	109±5	120±8	98±6	122±10
Diastolic BP (mmHg)	88±4	89±4	79±5	91±4
Cardiac Output (mL/min)	21.83±1.20	18.87±1.39	18.74±1.22	18.00±1.96
ESP (mmHg)	90.64±2.91	96.62±5.65	87.93±3.55	97.15±6.56
EDP (mmHg)	6.33±0.51	7.62±1.03	5.48±0.65	8.38±0.89
ESV (µL)	26.76±3.80	28.13±3.37	27.68±3.91	18.03±2.97
EDV (µL)	114.81±5.77	127.37±5.94	130.76±8.37	103.14±11.28
Stroke Volume (µL)	99.18±5.20	107.38±5.49	102.07±5.27	92.72±6.06
Ea (mmHg/µL)	0.95±0.06	0.95±0.08	0.89±0.06	1.08±0.12
.±dp/dt (mmHg/s)	6817.38±366.47	5818.13±305.96	5808.83±318.09	5466.00±230.07
.-dp/dt (mmHg/s)	-5215.69±314.67	-4227.13±256.39	-4173.17±263.75	-4565.17±205.80
Ejection Fraction (%)	75.91±2.42	75.35±1.87	73.79±2.25	79.59±1.38
**Occlusion Parameter**	**YC (n=13)**	**OC (n=10)**	**O+SVF (n=12)**	**O+BM (n=6)**
Emax (mmHg/µL)	2.09±0.25	1.73±0.19	1.87±0.24	1.19±0.13
EDPVR slope (mmHg/µL)	2.12±0.39	1.46±0.31	1.06±0.25	3.35±0.67
PRSW slope	104.51±9.38	79.54±3.60	81.13±3.95	78.76±7.59
PRSW intercept	-2127.74±865.62	-305.43±358.75	-1111.31±305.77	569.06±149.59 *

Data are presented as means±SEM, analyzed with one-way ANOVA followed by *post hoc* test where appropriate. *P* ≤ 0.05 vs Young Control (*), vs Old Control (#), and vs Old+SVF ($)

### Echocardiography and hemodynamic measurements

#### Echocardiography

To determine whether a cell therapy (SVF or BM-MSC) had any effect on cardiac parameters in aged animals compared to control, echocardiography was performed during rest and summarized in Table 2. OC rats exhibited a slight increase in stroke volume (SV) due to increased LV end systolic and diastolic volumes (LVVs/d) and dimensions (LVDs/d), but ejection fraction (EF) and CO were preserved (Table 2). The O+SVF (2.58±0.44, n=10) and O+BM (3.32±0.47, n=6) treated groups showed a significant increase in LVDs when compared to OC (2.17±0.33, n=9, *P*≤0.05) but only the SVF treated group had a significant increase in LV mass (500.49±46.39, n=10) when compared to YC (418.92±46.44, n=10) (Table 2). Both cell treated (O+SVF and O+BM) groups demonstrated a statistical difference when compared to YC in the following parameters: LVDs, LVDd, LVVs, and LVVd (Table 2).

The O+BM group had a significant decrease in EF and fractional shortening (FS) when compared to YC (Table 2). Additionally, the O+BM group had significantly reduced SV, EF, and FS compared to OC and O+SVF (Table 2).

#### Hemodynamic measurements

To determine whether a cell therapy (SVF or BM-MSC) had any effect on hemodynamic variables in aged animals compared to control, PV loop experiments were performed and summarized in Table 2. There were no significant differences regarding a majority of the PV loop measurements. This is likely due to the differential anesthetic effects of ketamine used for the PV loop measurements (ketamine is a known cardiac depressor [26]) vs. isoflurane used during echocardiography. During periods of vena cava occlusion, preload recruitable stroke work (PRSW) was measured as an index of myocardial contractility. The O+BM (569.06±149.59, n=6) group showed a significant loss in PRSW when compared to YC (-2127.74±865.62, n=13) (Table 2). Notably, there were no differences in PRSW between the O+SVF (-1111.31±305.77, n =12) treated group and YC (-2127.74±865.62, n=13).

#### Coronary flow reserve

**Figure 1:**
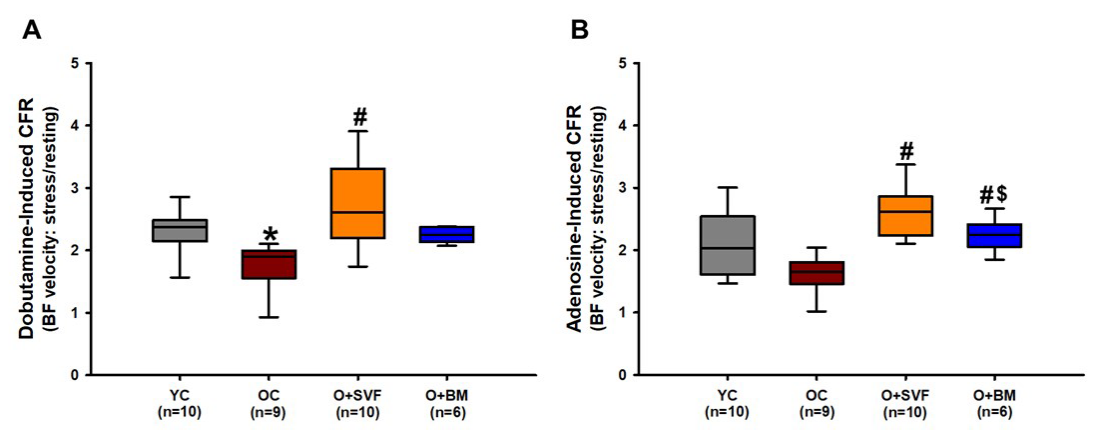
**Coronary flow reserve using Doppler echocardiography in rats.** Stress test was performed on experimental groups using dobutamine (**A**) or adenosine (**B**) and CFR was calculated. O+SVF group exhibited increased CFR vs. OC in both adenosine and dobutamine conditions. *P*≤0.05 vs Young Control (*), vs Old Control (#), and vs Old+ SVF($); Data are presented as means±SD, analyzed with one-way ANOVA followed by post-hoc Dunn’s (A) or Holm-Sidak (B) test.

In order to evaluate the ability of the heart to respond to a stressor, Doppler recordings of peak left anterior descending (LAD) artery BF velocity were measured during a dobutamine or an adenosine infusion (Fig 1A and 1B). The OC (1.77±0.4, n=9) group had diminished dobutamine-induced CFR compared to YC (2.31±0.36, n=10) (Fig 1A). Treatment with SVF therapy (O+SVF) significantly improved dobutamine- (2.7±0.68, n=10) and adenosine- (2.6±0.41, n=10) induced CFR when compared to OC (dobutamine 1.77±0.4; adenosine 1.61±0.26, n=9), achieving levels similar to YC (dobutamine 2.31±0.36; adenosine 2.08±0.55, n=10) (Fig 1A and 1B). Adenosine-induced CFR was significantly lower in the O+BM (2.27±0.27, n=6) group when compared to O+SVF treated, but was still significantly higher than OC (Fig 1B).

#### Diastolic function

**Figure 2:**
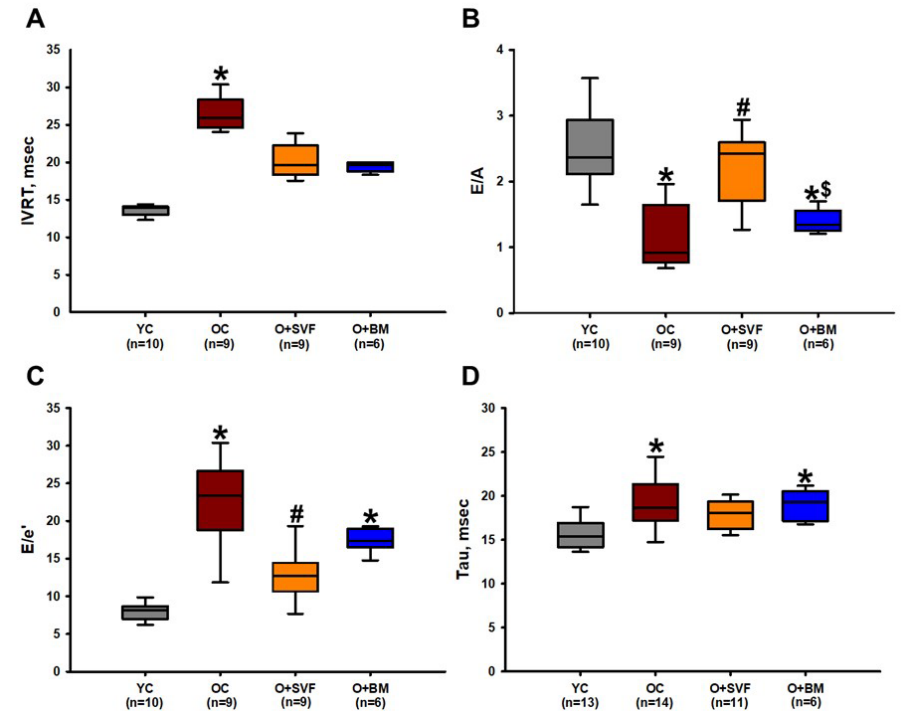
**Diastolic function assessment using echocardiography and pressure- volume loop (PV-loop).** Compared to YC rats, there was an age-related deterioration in diastolic function as measured echocardiographically by IVRT (**A**), E/A ratio (**B**), E/e’ ratio (**C**), and hemodynamically by Tau (**D**). Old rats treated with SVF significantly reversed this dysfunction in measures of E/A ratio (B) and E/e’ ratio (C) compared to OC, and normalized diastolic function to YC levels in IVRT (A) and Tau (D). *P*≤0.05 vs Young Control (*), vs Old Control (#), and vs Old+SVF ($); Data are presented as means±SD, analyzed with one-way ANOVA followed by post-hoc Holm-Sidak (B, D) or Dunn’s (A, C) test.

Echocardiographic measures of diastolic function are displayed in Figure 2, including isovolumic relaxation time (IVRT), E/A ratio – representing the ratio of peak velocity of blood flow from gravity in early diastole (the E wave) to peak velocity flow in late diastole caused by atrial contraction (the A wave) – and E/e’ – representing the ratio of mitral peak velocity of early filling (E) to early diastolic mitral annular velocity (e'). All measures showed an age-related deterioration in diastolic function when compared to YC (Fig 2A-D). The O+SVF treated rats showed a statistically significant improvement in E/A and E/e’ ratios when compared to OC (Fig 2B and 2C). The O+BM group was similar to OC in that it had significantly decreased E/A and E/e’ ratios as compared to YC, and E/A when compared to O+SVF (Fig 2B and 2C). There is a significant increase in the time constant of left ventricular relaxation measured by PV relationship (tau) when comparing OC (19.16±0.83, n=16) and O+BM (19.01±0.74, n=6) to YC (15.61±0.48, n=13) (Fig 2D).

### Subepicardial arteriole isolation experiments.

Arterioles under 150 μm in luminal diameter from the LAD distribution were dissected and isolated to assess whether cell therapy or adrenergic inhibition altered basic vessel characteristics such as spontaneous tone. Neither age (OC) nor a cell therapy (O+SVF and O+BM) significantly altered the maximum dimeter or average spontaneous tone reached (Table 3). Average tone after incubation with ICI118551, CPG20712A, or both ICI118551+CPG20712A was not significantly different between the groups or when compared to pre-inhibition (Table 3). β-AR agonists and antagonists were used to assess if cell therapy altered microvascular reactivity to adrenergic stimuli.

**Table 3:** Isolated vessel characteristics.

**Isolated Vessel Characteristics**	**YC (n=31)**	**OC (n=38)**	**O+SVF (n=32)**	**O+BM (n=16)**
Maximum Diameter	141.1±5.99	144.97±4.61	144.56±4.62	151.19±11.4
Average % Tone	28.77±5.51	29.59±1.19	30.1±1.73	25.87±1.83
Average % Tone after Incubation with ICI118551 (n)	25.43±1.2 (5)	31.05±2.94 (7)	27.04±2.69 (8)	29.45±15.57 (4)
Average % Tone after Incubation with CPG207212A (n)	26.94±2.26 (9)	29.52±1.97 (14)	34.25±3.59 (9)	38.42±4.05 (6)
Average % Tone after Incubation with ICI118551 + CPG207212A (n)	27.86±1.7 (9)	27.24±2.22 (9)	32.78±4.47 (6)	30.78±11.68 (5)

Luminal diameters were measured. Tone was calculated as a percent of the maximum diameter for pre- and post-incubation with an inhibitor. *P* ≤ 0.05 vs Young Control (*), vs Old Control (#), and vs Old+SVF ($); data are presented as means±SEM (n) and analyzed with paired t-tests for within group difference while between group difference were analyzed using one-way ANOVA

#### Vasodilation to dobutamine (β1-AR) and salbutamol (β2-AR)

**Figure 3:**
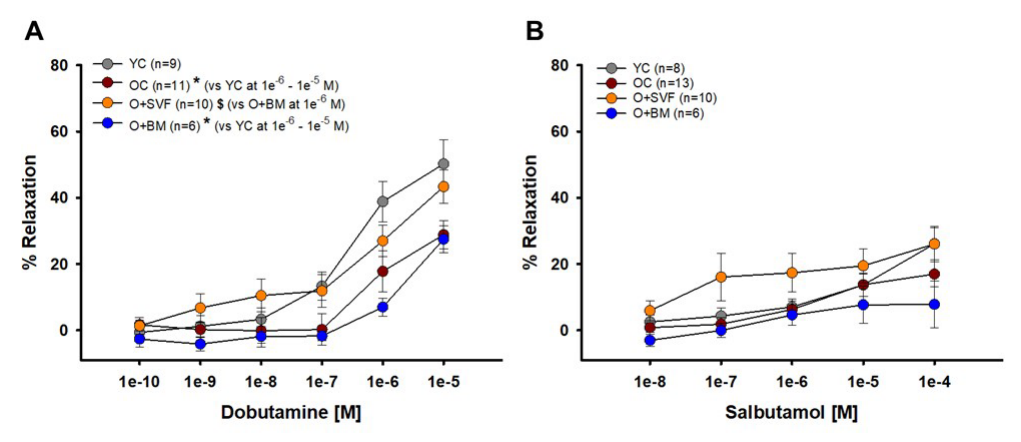
**Isolated coronary arteriolar vasoreactivity to β1- and β2-AR agonists.** Dobutamine, primarily a β1-AR agonist, induced vasorelaxation in all groups (**A**). Coronary arterioles from YC animals exhibited a significantly greater dilation compared to OC and O+BM at concentrations 1e^-6^ and 1e^-5^ [M] (*) (A). Salbutamol, a β2-AR agonist, induced mild vasorelaxation that was similar between all the groups (**B**). *P*≤0.05 vs Young Control (*), vs Old Control (#), and vs Old+SVF ($); data are presented as means±SEM and analyzed with two-way repeated measures ANOVA followed by post-hoc Bonferroni test.

Dobutamine and salbutamol were used to examine if cell therapy alters β1- (dobutamine) or β2-AR (salbutamol) responsiveness; data is represented in Figures 3 A and B. At the highest concentrations (1e^-6^ and 1e^-5^ [M]), vasorelaxation to dobutamine was significantly lower with age (OC) and in the O+BM group when compared to YC. At concentration 1e^-6^ [M], O+SVF (26.96±4.7, n=11) vasorelaxation was significantly higher when compared to O+BM (6.98±2.74, n=6) (Fig 3A). There was no difference between the groups at any dose of salbutamol; however, it is worth noting that the O+SVF group had a larger relaxation response at every dose compared to the other groups (Fig 3B).

#### Isoproterenol-induced vasodilation (non-selective β-AR agonist)

**Figure 4:**
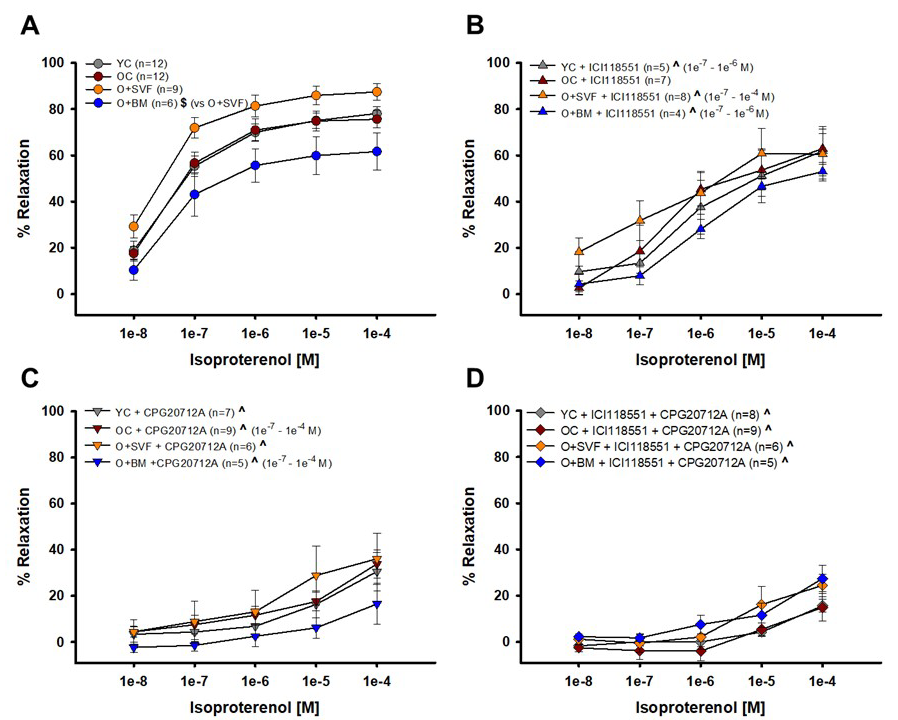
**Contribution of β1- and β2-AR to isoproterenol-induced vasodilation from isolated coronary arterioles.** Vasorelaxation to isoproterenol, primarily a non-selective β1-, β2-, and β3-AR agonist, was significantly impaired in the O+BM group compared to O+SVF ($) (**A**). Isoproterenol with ICI118551, a β2-AR antagonist, eliminated differences between the groups. Compared to pre-incubation, all groups except OC (YC, O+SVF, and O+BM) had significant attenuation in the inhibited dose response (^) at several concentrations (**B**). Isoproterenol with CPG20712A, a β1-AR antagonist, also eliminated differences between the groups, and all groups exhibited significant attenuation in the response compared to pre-inhibition (^) (**C**). No group differences to isoproterenol were noted following inhibition with both ICI118551 and CPG20712A, and all groups exhibited significant attenuation in the response compared to pre-inhibition (^) at every concentration (**D**). *P* ≤ 0.05 vs Young Control (*), vs Old Control (#), vs Old+SVF, and pre- vs post-inhibition (^); data are presented as means±SEM and analyzed with two-way repeated measures ANOVA, paired for inhibitor analysis, followed by post-hoc Bonferroni test.

Isoproterenol alone or combined with a β1- or β2-AR antagonist (or both) was used to examine whether receptor activity pre- and post-inhibition is altered with age, and if receptor activity is restored with cell treatment; this data is summarized in Figures 4 A-D. The complete concentration response of isoproterenol-induced relaxation in O+BM was significantly lower when compared to O+SVF (*P*≤0.005) (Fig 4A). Pre-incubation with ICI118551 (a β2-AR antagonist) revealed no difference between the groups. There was a statistically significant reduction in isoproterenol relaxation after inhibition with ICI118551 when compared to the uninhibited isoproterenol dose response for the YC, O+SVF, and O+BM groups at various doses (^) (Fig 4B). Preincubation with CPG20712A (β1-AR antagonist) revealed no difference between the groups (Fig 4C), but there was a significant attenuation of the 1e^-8^ [M] response in YC and O+SVF compared to their isoproterenol response (^). For concentrations 1e^-7^ through 1e^-4^ [M], all four groups showed a significant attenuation in relaxation when compared to their corresponding pre-inhibitor isoproterenol concentration response (^) (Fig 4C). Figure 4D used both ICI118551 and CPG20712A to show potential β3-AR activity. There was no difference between the groups, and every group showed a significant attenuation in relaxation when compared to the corresponding isoproterenol concentration response (^) (Fig 4D).

#### Norepinephrine-induced vasodilation (β1-, β2-, α1-, α2-AR agonist)

**Figure 5:**
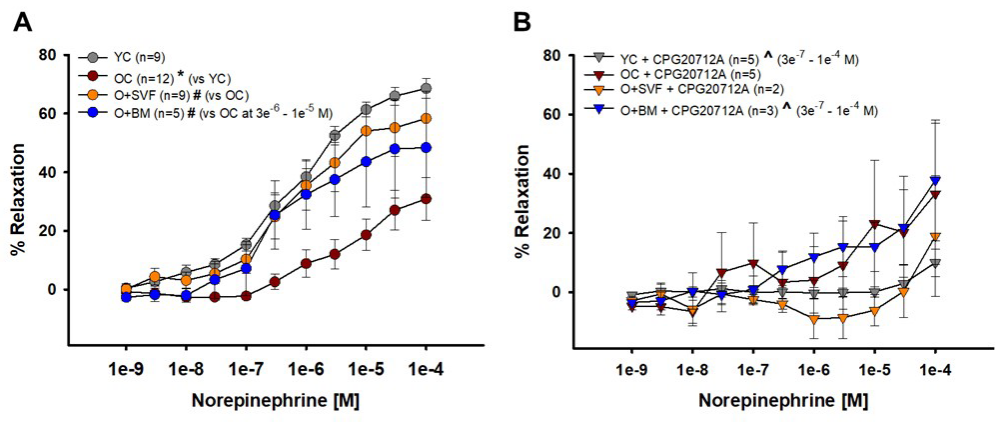
**Contribution of β2-AR to norepinephrine-induced vasoreactivity in isolated coronary arterioles.** Relaxation to NE was significantly impaired in OC animals compared to YC and O+SVF groups at all concentrations (**A**). NE with CPG20712A, a β1-AR antagonist, attenuated (^) the majority of the vasodilation response (3e^-7^ – 1e^-4^ [M]) in YC and O+BM treated groups (**B**). *P*≤0.05 vs Young Control (*), vs Old Control (#), vs Old+SVF ($), and pre- vs post-inhibition (^); data are presented as means±SEM and analyzed with two-way repeated measures ANOVA, paired for inhibitor analysis, followed by post-hoc Bonferroni test.

NE and the β1-AR antagonist CPG20712A were used to show vasoreactivity to a circulating catecholamine; data is represented in Figures 5 A and B. NE-induced relaxation was significantly decreased in the OC group compared to YC and O+SVF (Fig 5A). It is notable that there was no difference between YC and the O+SVF groups. When CPG20712A was used to attenuate NE-induced relaxation, there was a significant reduction in relaxation post-inhibition at concentrations 3e^-7^ through 1e^-4^ [M] for YC and O+BM (^) (Fig 5B).

### Immunofluorescence staining

**Figure 6:**
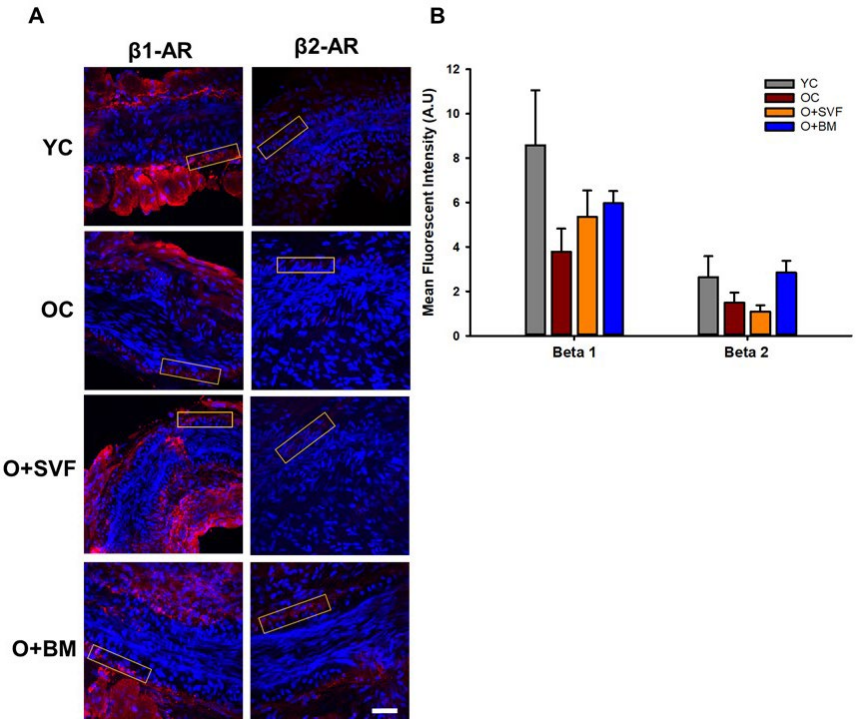
**β1- and β2-AR immunofluorescence in isolated coronary arterioles.** Representative images with ROI boxes used for fluorescent intensity analysis are shown on isolated coronary arterioles stained for β1- or β2-AR (**A**). There is no significant difference between the groups in the expression of β1- or β2-AR as measured by quantification of fluorescent intensity (**B**). Data are represented as means+SEM and analyzed with one-way ANOVA (n≥4). Scale bar is 50 micrometers.

To assess changes in populations of β1- or β2-AR with advancing age or cell therapy treatment, immunofluorescence was performed on a subset of microvessels from explanted hearts. Representative images from each group were used to show the fluorescence intensity measured on the endothelial-vascular smooth muscle wall of each microvessel (Fig 6A). Quantification of β1- or β2-AR fluorescence is shown in Figure 6B. Although not statistically significant, there was a decrease in both the β1- and β2-AR with advancing age. Both cell therapies increased levels of β1-AR compared to OC, while treatment with BM-MSC, but not SVF, increased levels of β2-AR.

## DISCUSSION

The major finding from this study is that aged rats treated with intravenous SVF exhibit a restoration of cardiac β-AR responsiveness compared to untreated old controls. This was realized four weeks following SVF treatment by improved CFR, diastolic function, and coronary microvascular reactivity to adrenergic agonists, isoproterenol and norepinephrine. Part of our original hypothesis was supported by the findings in this study, as both SVF and BM-MSC cell therapies were associated with improved arteriolar vasodilation to NE (a non-selective adrenergic agonist), but the BM-MSC group did not show improvements in CFR, diastolic function, or vasodilation to isoproterenol or dobutamine as described in the O+SVF group.

Although still controversial [27], the majority of evidence supports a beneficial effect of cell therapy in treating ischemic and/or dysfunctional cardiac tissue, and this is likely due to still not well-defined paracrine factors released by or in response to the injected cells. Previous studies have shown that MSCs and adipose-derived SVF can be anti-inflammatory and immune-modulatory [28,29], possibly via influence from the sympathoadrenal system [30]. Additionally, our past studies have shown that SVF cells can incorporate into the vascular wall long-term [23] and are associated with normalization of inflamed large vessels, possibly via alteration in reactive oxygen species (ROS) signaling [31]. It’s well known that HF leads to enhanced release of catecholamines [32,33] and these levels are correlated to the severity of HF [34], resulting in a desensitization of β-ARs which can ultimately promote more cardiac dysfunction [33]. Catecholamine excess has also been demonstrated in advancing age [35], shown to be partially consequent on ROS generation [36], and inversely linked to vascular endothelial function [37]. In 2006, Dhein and colleagues [24] demonstrated for the first time that intramyocardial injection of BM-MSC normalized both circulating catecholamine levels and β-AR density in the heart after four weeks in a model of non-ischemic cardiomyopathy in rabbits. Although this wasn’t statistically significant, our data supports this, as we show an age-related increase in plasma catecholamines that was decreased following BM-MSC or adipose-derived SVF injection (Table 1).

BM-MSCs were initially hailed as the superior autologous cell source to treat myocardial infarction (MI) because of their differentiation potential [38,39], but recent studies have shown equal efficacy of adipose-derived SVF in improving cardiac function after MI and *in vitro* induction into cardiomyocyte-like-cells and vascular elements [40–43]. The present study shows that SVF but not BM-MSC therapy improved diastolic function in aged rats, as measured by E/A, E/e’, and tau (Fig 2B-D). It is worth mentioning that some group differences observed by ultrasound (EF and SV) were not present for hemodynamic outcomes, but this is likely due to the use of ketamine anesthesia during hemodynamic measurements, which has been associated with depressed LV function (Table 2) [26]. Regardless, our data stands in contrast to the Monnerat-Cahli [25] study where 5x10^6^ BM-MSCs improved left ventricular developed pressure (LVDP) four weeks after injection in a diabetic rat model. Although we purposefully utilized the same concentration and timeline of BM-MSC treatment in the present study, we did not observe improved cardiac function in the O+BM group compared to the O+SVF group (Fig 2 B-D), possibly due to the streptozotocin-model of diabetes, use of young male Wister rats, or the method of LVDP measurement in an isolated working heart as performed by Monnerat-Cahli and colleagues [25].

Dobutamine and adenosine both increase cytosolic cAMP production [44] in the coronary circulation to achieve hyperemic flow, but this occurs through different receptor activation (β-AR and adenosine receptors, respectively). The age-related decrease in dobutamine-induced CFR was significantly reversed after SVF treatment (Fig 1A), indicating SVF cell therapy improved cardiac β-AR responsiveness. Although age-related deficits were not revealed during adenosine-induced CFR, both cell therapies (SVF and BM-MSC) significantly improved CFR compared to OC (Fig 1B). These disparate results may be explained by the fact that β-AR-mediated accumulation of cAMP has been shown to decrease with age, but alternate induction pathways of cAMP appear to be preserved. To this end, relaxation to forskolin - a direct activator of adenylyl cyclase (AC) - are normal in whole vessels [45] while basal levels of AC are increased with age in rat aorta [46], suggesting that the AC enzyme and events distal to cAMP formation are intact with age. Ross et al. [47] showed *in vivo* that infusion of propranolol (a β1- and β2-AR inhibitor) decreased peak coronary blood flow in response to isometric handgrip exercise in young men, whereas old men (67±4 years) exhibited no effect. These studies support that SVF cell therapy in aged hearts increase CFR through improvements in β-AR-cAMP signaling.

We found that there is a significant age-related decrease in β1-AR-mediated vasodilation, as YC demonstrated increased vasodilation to dobutamine (Fig 3A) vs OC. In this study, we believe SVF therapy worked by improving β-AR, specifically β1, in the coronary microcirculation because vasodilation to dobutamine was increased following SVF treatment vs the O+BM group. Further, when β1-AR was inhibited using CPG20722, age- and group-related differences were eliminated in the vasodilation to NE (Fig 5B). Dobutamine can elicit differential effects depending on the tissue; in the cardiac tissue it acts mostly via β1-AR stimulation versus in the vascular system it stimulates β1- and β2-AR vasodilation, and also some minor α1-AR vasoconstriction [48]. But the importance of β1-AR stimulation prevails, as Abdelkrim and colleagues [49] demonstrated that immunization against the β1-AR in young rats produced significant decreases in dobutamine and isoproterenol relaxations in small isolated mesenteric arteries, and impaired endothelial-dependent nitric oxide (NO) signaling pathways. As mentioned previously, there is decreased β-AR responsiveness with age [50] and in HF [8,33] and our findings support this conclusion.

Pretreatment with ICI118551, a β2-AR antagonist, abolished the difference in vasodilation to isoproterenol between the cell-treated groups (Fig 4 A&B), suggesting that there is some contribution of β2-AR activity and/or signaling following SVF but not BM-MSC cell therapy. This link between SVF treatment and heightened β2-AR activity is supported by the age-related decrease in relaxation to NE in coronary arterioles, which was restored in the SVF cell-treated group (Fig 5A). Historically, NE effects on adrenergic receptors have been difficult to study because of the multifaceted/opposing effects on vascular and cardiac tissues. Vasodilation to NE has been previously shown to be primarily mediated through β2-AR on the VSMC of human coronary arterioles [51]. However, we did not find a significant difference in vasodilation between the groups using the selective β2-AR agonist, salbutamol (Fig 3B). While β2: β1-AR ratio increases in failing human hearts [32] and in senescent hearts [3,52], we have a novel observation that the opposite occurs in β2-AR staining in arterioles from OC, and this wasn’t altered with SVF treatment (Fig 6).

If β1-AR undergoes prolonged stimulation, similar to chronic catecholamine spillover in the elderly [53], β2-AR can undergo a cross-desensitization [35]. Schutzer et al. [54] suggested that β2-AR actually achieves maximal desensitization with age, resulting in β2-AR switching from G_stimulatory_ to G_inhibitory_. To that end, β2-AR stimulation with concomitant β1-AR inhibition has been shown to improve cardiac function and myocardial O_2_ consumption post-operatively in aged male rats [55]. Of note, β2- and β3-AR are functionally distinct from β1-AR by their ability to deactivate ACs by coupling to the inhibitory G protein (Gαi). Cardiac Gαi levels and activity have been shown to be increased with age in humans and in rodent models [56–58]. It’s likely that aging may have a direct influence on β2-AR activity through indirect receptor cross-desensitization due to circulating catecholamines and/or an increase in Gαi activity, and that SVF cell therapy reverses this, but further studies are required to explore this possibility.

It’s important to consider the limitations of the present study: 1) radioligand binding would be a more direct method of measuring β1- and β2-AR density and ratio in isolated coronary arterioles compared to our immunofluorescence method, 2) catecholamine levels were analyzed in plasma collected at the conclusion of PV loop experiments while anesthetized with ketamine. Ketamine has been proven to decrease plasma catecholamines whilst inhibiting the uptake of NE, resulting in a transient increase [59]. This may have caused a dampening in the true catecholamine levels. 3) In order to be consistent with a previous study [25], the concentration of cells between the two cell groups were not the same. 4) This study only utilized female rats as recipients. Our rationale for this is because as age progresses in humans, CVD and CMD manifests in the sexes differently. As many as 50% of women referred for evaluation of MI do not have obstructive coronary disease, like most men, but are frequently associated with coronary microvascular dysfunction/ischemia [60]. Follow-up studies suggest that CFR is a better predictor of future adverse events compared to traditional angiographic methods in older women [61]. 5) The present study utilized SVF and BM-MSC from young donor rats only. Our lab and others have previously shown that the age of the donor significantly depresses the angiogenic, vasculogenic, and overall mesenchymal potential of isolated cells [62–64], but it’s unknown whether these factors would alter the outcomes in the present study. Lastly, β3-AR plays a key role in the development of LV diastolic dysfunction as recently shown by Yang et al. using a knockout mouse model [65]. The role of β3-AR wasn’t directly explored in the current study but warrants investigation to determine if aging and/or cell therapy alter its activity and/or expression.

Our previous study showed SVF incorporation into the cardiac and vascular space four weeks post-injection in an aged rat [23]. The field is still unsure if the beneficial effects of injected cells are mediated via secreted factors being released from the cells or if it is the incorporated cells themselves remaining in the circulation and populating the peripheral tissue [66]. Regardless, this new data provides exciting evidence that SVF cell treatment can improve vasodilation to adrenergic agonists and increase CFR compared to old controls rats, providing a new strategy in managing sympathetic desensitization that occurs in advancing age as well as diastolic dysfunction. These results lay significant groundwork into future studies on cell therapy and resultant sympathoadrenal and β-AR signaling.

## MATERIALS AND METHODS

### Animal model, groups, and endpoint procedures

All animal surgeries were performed in accordance with protocols approved by the University of Louisville Institutional Animal Care and Use Committee and the NIH *Guide for the Care and Use of Laboratory Animals* [67]. Young (3 mo) and old (22 mo) female Fischer-344 rats (Harlan Laboratories, Indianapolis, IN, USA and National Institute on Aging, Bethesda, MA, USA, respectively) were housed in groups with free access to food and water and were maintained on regular 12-hour light/dark cycles. Rats were acclimated to facility conditions for a minimum of one week prior to endpoint procedures. The remaining old rats were divided randomly into two cell injection groups: old + GFP+ bone marrow stem cells (O+BM) and old + GFP+ SVF cells (O+SVF). Four weeks later, old cell-injected rats were 23-24 months of age at endpoint. All animals were utilized for PV loop data acquisition as well as isolated coronary arteriole experiments. A subset of animals from each group were utilized for echocardiography and immunofluorescence. Animals were anesthetized deeply with a ketamine/xylazine injection before euthanasia via removal of the heart.

#### Rat bone marrow cell isolation and culture

BM-MSC were isolated from femur and tibia bones of young female and male Fischer-344 GFP+ rats (colony maintained in house) using a protocol modified from Barbash et al. [68] and Lennon et al. [69]. Briefly, 3-4 donor animals were anesthetized deeply with 5% inhaled isoflurane balanced with 1 L/min O_2_ and then euthanized via removal of the heart. Using sterile technique, femur and tibia bones were exposed and disarticulated at the associated joints, and extraneous muscle and tissue removed leaving only the bones of interest. Care was taken to obtain whole intact bones from each animal. After removal of muscle and connective tissue, bones were stored in DMEM + Penicillin/Streptomycin on ice. Bone cutters were used to remove the proximal and distal end from each bone just below the marrow cavity. Whole bone marrow was then flushed with 10mL sterile Complete Medium (DMEM + 15% fetal bovine serum + Glutamine + Penicillin/Streptomycin) per bone using a 10 mL syringe and 27g needle; bone marrow was collected into a sterile 70 µm filter screen fitted onto a sterile 50 mL conical tube. After filtration, cells were centrifuged at 1400 rpm for 8 min at room temperature and counted with a hemocytometer. Freshly isolated BM-MSC were cultured with an initial seeding density of 7.5x10^7^ cells per T75 culture flask in 10 mL Complete Medium; flasks were placed in a 37°C incubator with 5% CO_2_ for 3 days, after which medium was changed every 2-3 days until 95% confluence. Cells were then treated with 0.25% trypsin at 37°C for 2 minutes and passaged into T75 culture flasks (1.5x10^6^ cells/flask). Media was changed every 2-3 days and flasks passaged at 95% confluence; this method was repeated until cells were harvested at P3 or P4 for infusion into O+BM rats.

#### SVF isolation

SVF cells were isolated from a GFP+ Fischer-344 rat colony (maintained in house) as previously described [23,70]. Briefly, ovarian or epididymal fat pads from young female and male rats respectively (3-6 mo) were harvested, washed, finely minced, and digested in 0.75 mg/mL Type I collagenase solution (Vitacyte). Buoyant adipocytes were removed via centrifugation, and the SVF cell pellet resuspended in 0.1% BSA-PBS as previously described [23,70]. Freshly isolated SVF cells were then prepared for injection into O+SVF rats.

#### Cell injections

BM-MSC populations were trypsinized. BM-MSC and SVF isolate were then washed and filtered separately through a 20 µm screen to eliminate large cell and tissue aggregates. Cell count was determined with a hemocytometer, and GFP+ fluorescence of the cell population confirmed via fluorescence microscopy prior to injection. Old female rats were randomly divided into the two cell injection groups, then injected intravenously with either 10^7^ GFP+ SVF cells or 5x10^6^ GFP+ BM-MSC in 1 mL lactated Ringers solution (warmed to 37°C) via the tail vein.

### Echocardiography

#### Systolic and diastolic parameters

LV systolic and diastolic function were evaluated by transthoracic echocardiography using a Vevo 3100 with MS250D transducer with a frequency of 13-24 MHz as previously described [23] (FUJIFILM VisualSonics Inc., Toronto, Ontario, Canada). Briefly, rats were anesthetized and maintained with isoflurane (induction chamber at 5% with 1.5–2.0 L/min O_2_ flow followed by 1.5% with 1.5 L/min O_2_ flow). Rats were then placed in a supine position and the thorax was shaved. Body temperature was maintained at 37–38°C, and heart rate was monitored using Vevo Imaging Station. Variables that represent diastolic function - IVRT, E/A and E/e’ ratios - were obtained during resting condition utilizing an apical four chamber view with conventional pulsed wave Doppler and tissue Doppler. E/A ratio was calculated from the peak velocity flow in early diastole (the E wave) to peak velocity flow in late diastole caused by atrial contraction (the A wave) during resting conditions [23]. Results from five cardiac cycles during expiration were averaged together and used for between-group and within-group comparisons.

#### Coronary flow reserve measurements

In addition to the standard echocardiographic imaging of cardiac function, a modified parasternal short-axis projection was used for Doppler recording of the LAD during rest while animals were anesthetized with 1.5% inhaled isoflurane, and again during two cardiac stress states induced via dobutamine (20 µg/kg/min) and adenosine (140 µg/kg/min) in a random order. The tail vein was cannulated with a 27-gauge butterfly needle for drug administration. Dobutamine and adenosine were infused for a maximum of 5 minutes using an automated perfusion pump (KD Scientific, Holliston, MA), with a recovery period between drugs to allow the heart to return to baseline LAD velocity and heart rate. LAD BF velocity pre-stress and during the stress challenge were averaged from three consecutive cardiac cycles and CFR was calculated as the ratio of the mean peak LAD BF velocity values during each stress condition and rest [71].

### Hemodynamic measurements

Rats were anesthetized with an intraperitoneal injection of ketamine (50 mg/kg) / xylazine (12.5 mg/kg) / acepromazine (2.0 mg/kg) mix. Conductance readings were made for ~35–60 min prior to harvesting heart tissue. Briefly, the rat was placed in a supine position on a 37 °C pad, tracheotomized and connected to a ventilator to control breathing. The right carotid artery was isolated using silk sutures. The cranial aspect of the carotid artery was ligated and a microsurgical clip was placed on the proximal carotid artery for hemostasis. The chest cavity was opened between the right 5^th^ and 6^th^ intercostal area and retracted to expose the inferior vena cava (IVC) near the diaphragm; saline-soaked silk suture was placed under the IVC and left for later use during occlusion measurements. An arteriotomy was performed with microsurgical scissors, and a 1.9 F PV 6.00mm conductance catheter (Transonic, London, ON, Canada) calibrated to current atmospheric pressure was introduced into the carotid artery. The catheter was then secured into the carotid artery with sutures and advanced retrograde across the aortic valve into the LV; continuous hemodynamic monitoring insured proper catheter placement in the LV. PV loops were recorded in the steady state with the ventilator off for 8-10 seconds to reduce loop variability. The ventilator was turned on immediately following baseline recording. After 2-3 minutes of recovery, the ventilator was again turned off and the silk suture under the IVC was gently lifted to induce occlusion of the IVC. Immediately following occlusion recording, catheter was removed and the animal was humanely euthanized via vital organ removal. Data recording and analyses of both baseline and occluded PV loops were performed using LabChart Pro software (ADInstruments, Colorado Springs, CO).

### Subepicardial arteriole isolation experiments

The heart was removed from each animal and coronary arterioles from the LAD artery distribution were isolated and transferred to a vessel chamber. Arterioles were then cannulated on both ends, pressurized to 45 mmHg [72], and allowed to develop spontaneous tone (> 20% constriction from initial diameter) [23].

The following experiments were randomized in each vessel. Concentration-response curves to isoproterenol (non-specific β-AR agonist, Sigma-Aldrich I6504), dobutamine (primarily a β1-receptor agonist with lower β2 activity, Sigma-Aldrich D0676), NE (α1-, α2-, β1-, and β2-AR agonist, Sigma-Aldrich A9512), and salbutamol (β2-AR agonist, Sigma-Aldrich S8260). Following the agonist responses, some vessels were incubated in the β1-receptor antagonist CPG20712A (Sigma-Aldrich C231) prior to a second concentration-response curve of isoproterenol or NE. Other vessels were incubated in the β2-receptor antagonist ICI118551 (Sigma-Aldrich I127) prior to a second concentration-response curve of isoproterenol. A third subset of vessels received both CPG20712A and ICI118551 simultaneously prior to an additional concentration-response curve of isoproterenol. Upon completion of all response curves, vessels were washed 2x15 min in Ca^2+^-free physiological salt solution followed by a single dose of sodium nitroprusside (1e^-4^ [M]). Maximum diameter was determined as the largest diameter achieved throughout the experiment. Tone was calculated as 1-(initial diameter/maximum diameter) x 100.

### Immunofluorescence staining

Coronary arterioles (750 μm-1 mm in length, <250 μm in diameter) were isolated and fixed in 2% paraformaldehyde in 48- well plates for 1-hour, washed 2x10 min in DCF-PBS, and placed in 0.5% Triton-X/DCF-PBS for 20 minutes at room temperature (RT). After treating with blocking solution for 1 hour at RT, arterioles were incubated in a primary β1-AR antibody (Abcam, ab3442, 1:150) or β2-AR (Abcam, ab182136, 1:100) solution in blocking solution at 4°C overnight with gentle rocking. Normal rabbit serum replaced primary antibody solution for negative control tissues. A donkey anti-rabbit IgG-Alexa Fluor 594 (Invitrogen, A21207, 1:300) blocking solution was added to the tissues and incubated for 1-hour at RT. Nuclei were stained with Dapi. Tissues were placed on slides with anti-fade mounting media, coverslip added, and imaged using a Nikon ECLIPSE confocal microscope system (Nikon, Tokyo, Japan) with a 405 and 562 nm laser. Images were captured at 1024X1024 pixel density and 2 µm Z-step (minimum 10 stacks) with a 40X oil immersion objective. Immunofluorescence intensity of β1- or β2-AR in the region of interest (ROI) boxes were determined by the Nikon NIS Elements AR Analysis software (Nikon) using 20 µmx100 µm area and at least 8 µm in depth [73]. ROI boxes were placed on the vascular wall overlapping both the endothelial and smooth muscle cell layers, but excluding the cardiomyocytes.

### Statistical analysis

Statistical analyses were performed with SigmaPlot 14 (Systat), and the significance level was set at *P*≤0.05. Animal characteristics, catecholamine levels, echocardiography and hemodynamic measurements, and immunofluorescence intensity were analyzed using one-way ANOVA followed by Holm-Sidak or Dunn’s *post hoc* tests where appropriate. When normality tests failed, one-way ANOVA on Ranks were performed where appropriate. Concentration-response comparisons for between group differences were analyzed with two-way repeated measures ANOVA. Pre- and post-inhibition data were paired for analysis and inhibition of the concentration-response comparisons for within group differences were analyzed with two-way repeated measures ANOVA. All significant interactions were further investigated using Bonferroni *post hoc* testing. Tone acquired pre- and post-inhibition were analyzed using a paired t-test for within group difference while between group difference were analyzed using one-way ANOVA. Data are represented as means±SD or SEM as indicated.
